# Thigh Muscle MRI Edema Features of Dermatomyositis Patients With Ovarian Cancer: A Report of Three Cases

**DOI:** 10.7759/cureus.24337

**Published:** 2022-04-21

**Authors:** Xiaoxiao Cheng, Qi Fang

**Affiliations:** 1 Neurology, The First Affiliated Hospital of Soochow University, Suzhou, CHN

**Keywords:** cancer risk, ovarian cancer, honeycomb edema signal, magnetic resonance imaging, dermatomyositis

## Abstract

Dermatomyositis (DM) is a rare inflammatory disease with systemic manifestations, including internal malignancy. Ovarian cancer is one of the most commonly found tumor types in DM patients. To date, no study has analyzed the MRI signals of dermatomyositis patients with cancer. Thus, in this report, we describe three cases in which we found ovarian cancer after the diagnosis of DM and observed a symmetrical honeycomb pattern edema signal distributed to the peripheral area of vastus laterals while adductors and posterior thigh compartments were relatively spared. Our report has important implications for clinicians to identify patients with dermatomyositis at potential ovarian cancer risk.

## Introduction

Dermatomyositis (DM) is a rare autoimmune disease characterized by skin lesions, muscle weakness, and other systemic manifestations [1], with an estimated prevalence rate of 1-6 per 100,000 adults [2]. Cancer is one of the major complications of DM that determines the prognosis of patients. Some researchers have even raised the question of cancer as a cause rather than a consequence of autoimmunity [3]. As a possible paraneoplastic disorder, DM is correlated with various types of cancer, including gastrointestinal cancer and nasopharyngeal cancer [4]. The significant relationship between underlying ovarian cancer and dermatomyositis has recently been studied [5-6], with a prevalence of 13.3% [5].

Musculoskeletal MRI is a widely used tool in clinical work to provide information ranging from the subcutaneous tissue, fascia, to muscle. The main functions of MRI are as follows: a. early diagnosis of CADM and fasciitis; b. differential diagnosis with other types of myopathies, such as congenital myopathy; c. locating the site for biopsy; and d. monitoring the disease activity and evaluation of treatment response with follow-up scans [7]. Typical MRI edema features like subcutaneous and fascial hyperintensity are considered valuable to identify inflammatory myopathies and their phenotypes [8]. Some typical MRI signals are also predictors of clinical outcomes (for example, subcutaneous fat signals) [9].

To date, no study has analyzed the MRI signals of dermatomyositis patients with cancer. In this report, we described three DM patients who were diagnosed with ovarian cancer and detailed their thigh muscle MRI findings.

## Case presentation

Case 1

**Figure 1:**
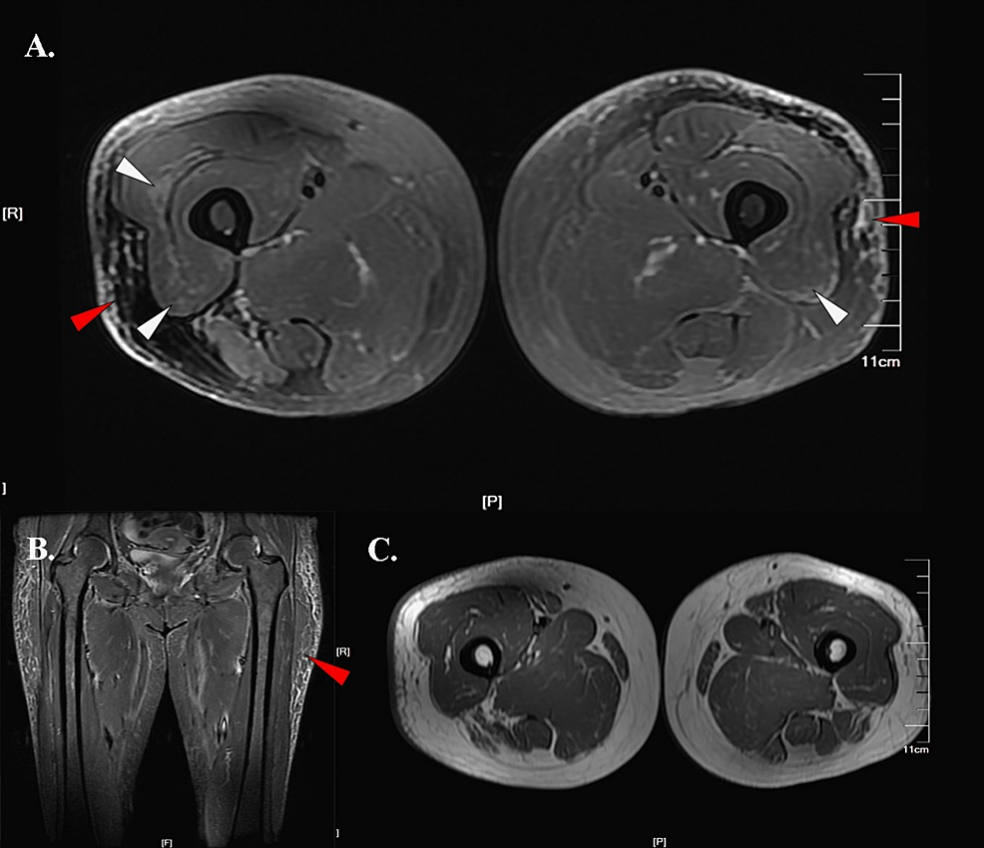
Thigh muscle MRI images of Case 1 A. An axial T2 fs sequence image showed subcutaneous adipose tissue involvement (red arrows) and peripheral honeycomb edema signal of the vastus lateralis (white arrows). B. A coronal STIR sequence image showed subcutaneous adipose tissue involvement (red arrow). C. An axial T1 sequence image showed minimum fatty infiltration. Abbreviations: T2 fs, T2-weighted fat saturation; STIR, short time of inversion recovery.

A 63-year-old postmenopausal female admitted to our hospital presented with skin rash and muscle weakness for two months. Physical examination demonstrated Gottron’s sign and heliotrope rash, with grade IV proximal muscle strength. The patient's muscle strength improved with treatment of intravenous methylprednisolone injection (80 mg/d for five days then 40 mg/d for five days) followed by oral prednisone intake. Her tumor biomarker was elevated (detailed information shown in Table 1). Thus, further examinations were performed. No obvious abnormality in the annex area was found with gynecological ultrasound. An enhanced CT scan of the chest, abdomen, and pelvis showed multiple significant lymphadenopathies in the right inguinal region and pelvis. The pathological result of right inguinal lymph node needle biopsy showed metastatic adenocarcinoma with necrosis, and ovarian cancer was first considered. With further confirmation by PET/CT, the diagnosis of ovarian cancer was made, and she was transferred to the oncology department for further chemotherapy treatment with cisplatin, endostatin, and paclitaxel liposome. Her thigh muscle MRI (Siemens, 3.0 T unit; Munich, Germany) revealed reticular hyperintensity of subcutaneous adipose tissue on T2 fs (T2-weighted fat saturation)/STIR (short time of inversion recovery) sequences (Figures 1A-1B). Fascia involvement was also observed. Specifically, the honeycomb/reticular pattern hyperintensity signal was found in the proximal peripheral area of the vastus lateralis (Figures 1A-1B) with minimum fatty infiltration (Figure 1C). No edema signal was found in the adductors and posterior thigh compartment.

**Table 1:** Clinical characteristics, laboratory findings, and treatment of the studied patients Y, yes; N, no; N.A., not available; (-), negative Abbreviations: WBC, white blood cell; CK, creatine kinase; LDH, lactate dehydrogenase; ALT, alanine aminotransferase; CRP, C-reactive protein; ESR, erythrocyte sedimentation rate; ANA, antinuclear antibody; MSAs, myositis specific antibodies; TIF1-γ, transcriptional intermediary factor 1; TSGF, tumor special growth factor; CA12-5, carbohydrate antigen 12-5; CA19-9, carbohydrate antigen 19-9; CA15-3, carbohydrate antigen 15-3; CEA, carcinoembryonic antigen; AFP, alpha-fetoprotein

Variable	Case 1	Case 2	Case 3
Basic information			
Gender	Female	Female	Female
Age of onset (year)	63	62	60
Body mass index	21.9	25.4	21
Clinical manifestation			
Disease duration (month)	2	3	6
Skin rash	Y	Y	Y
Muscle weakness	Y	Y	Y
Muscle/joint pain	N	N	N
Dysphagia	N	Y	Y
Interstitial lung disease	N	N	N
Laboratory test results			
WBC count (10^9/L)	8.62	5.11	5.89
Hemoglobin (g/L)	123	116	94
CK (U/L)	30.9	74.9	136.8
LDH (U/L)	245.4	401.5	308.8
ALT (U/L)	19.8	16.7	16
Serum ferritin (ng/ml)	285.49	175.32	456.12
CRP (mg/L)	0.26	3.76	15.36
ESR (mm/h)	10	81	120
ANA	(-)	(+)	(-)
MSAs	N.A.	TIF-1	N.A.
TSGF (U/ml)	72.50­	74.50	72.50
CA12-5 (U/ml)	29.30	915.10­	957.80­
CA19-9 (U/ml)	0.25	11.85	0.00
CA15-3 (U/ml)	24.60	54.00­	51.80­
CEA (ng/ml)	8.23	0.95	41.14­
AFP (mg/L)	1.36	3.75	1.55
Treatments			
Methylprednisolone (mg/d)	80	40	40
Immunosuppressant	N	N	hydroxychloroquine
Immunoglobulin	N	Y	N

Case 2

**Figure 2:**
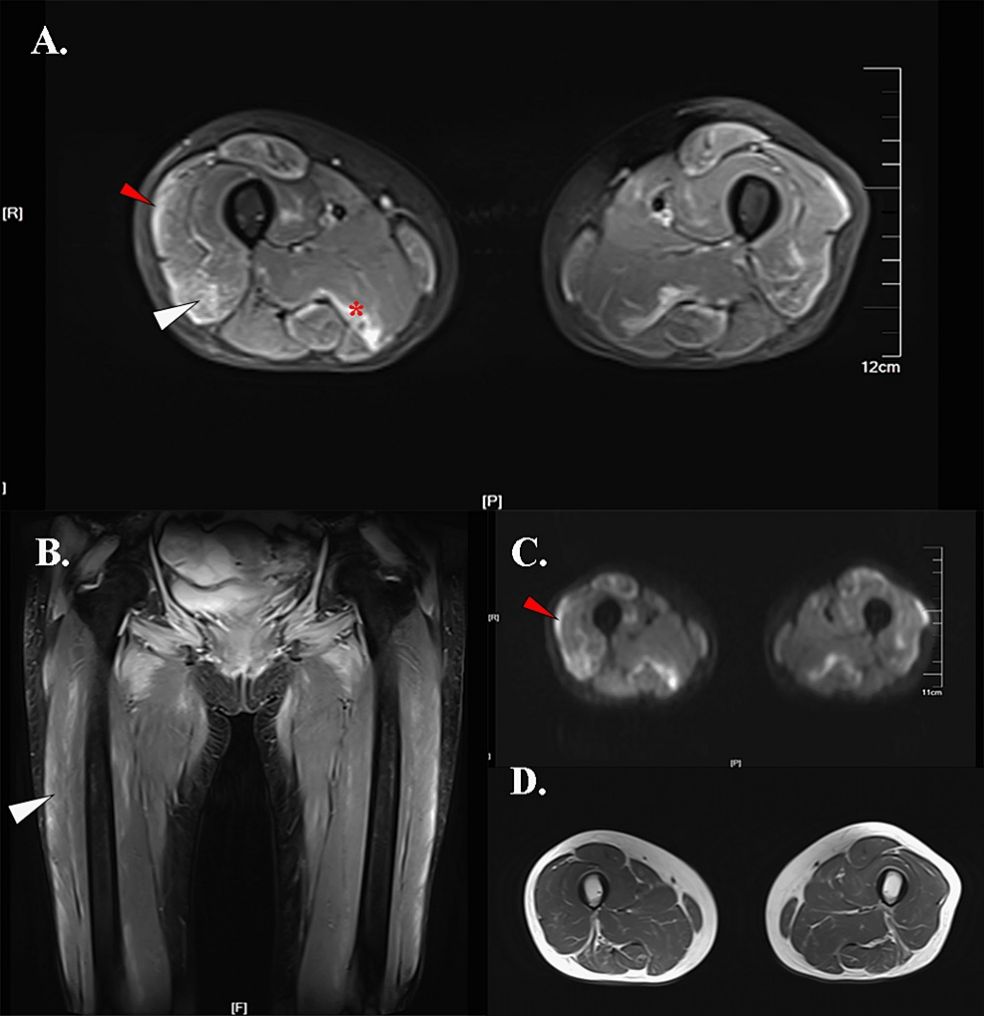
Thigh muscle MRI images of Case 2 A. A STIR sequence image showed fascia involvement (red arrow), peripheral honeycomb edema signal of vastus lateralis (white arrow), and peripheral foggy edema signal of the adductor magnus (red *). B. A coronal STIR sequence image showed a vastus lateralis edema signal (white arrow). C. An axial DWI sequence image showed a fascia hyperintensity signal (red arrow). D. An axial T1 sequence image showed no fatty infiltration. Abbreviations: STIR, short time of inversion recovery; DWI, diffusion-weighted imaging

A 62-year-old postmenopausal female patient with a history of autoimmune hepatitis developed muscle weakness in the proximal limbs for three months. The symptoms started worsening in the past month with difficulty walking, combing, head lifting, and swallowing. She was admitted to our department, and physical examination showed bilateral cheek erythema as well as grade III muscle strength for the proximal extremities. The myositis-specific autoantibody (MSA) test was positive for the anti-TIF-1 autoantibody. The patient's symptoms improved significantly after treatment with methylprednisolone (40 mg/d for seven days) and immunoglobulin (10 g/d for five days). During the patient's hospitalization, ultrasonography showed a solid mass in the left pelvis and a cystic mass in the right adnexal area, and enhanced CT showed cystic lesions in both adnexa with consideration of cystadenocarcinoma. The patient was transferred to the gynecology department for a needle biopsy showing poorly differentiated ovarian adenocarcinoma and then underwent a hysterectomy, ovariosalpingectomy, followed by chemotherapy with cisplatin and fluorouracil intraperitoneal injection. Combined with postoperative pathology, poorly differentiated serous adenocarcinoma was considered. Her muscle MRI showed bilateral fascia involvement (Figure 2A, Figure 2C) and a typical honeycomb edema signal distributed to the peripheral area of the vastus lateralis (Figures 2A-2B). The adductor magnus presented with a peripheral foggy edema signal, as shown in Figure 2A. We also observed severe involvement of the pelvic muscles. No adipose tissue edema and fatty steak were found (Figure 2D).

Case 3

**Figure 3:**
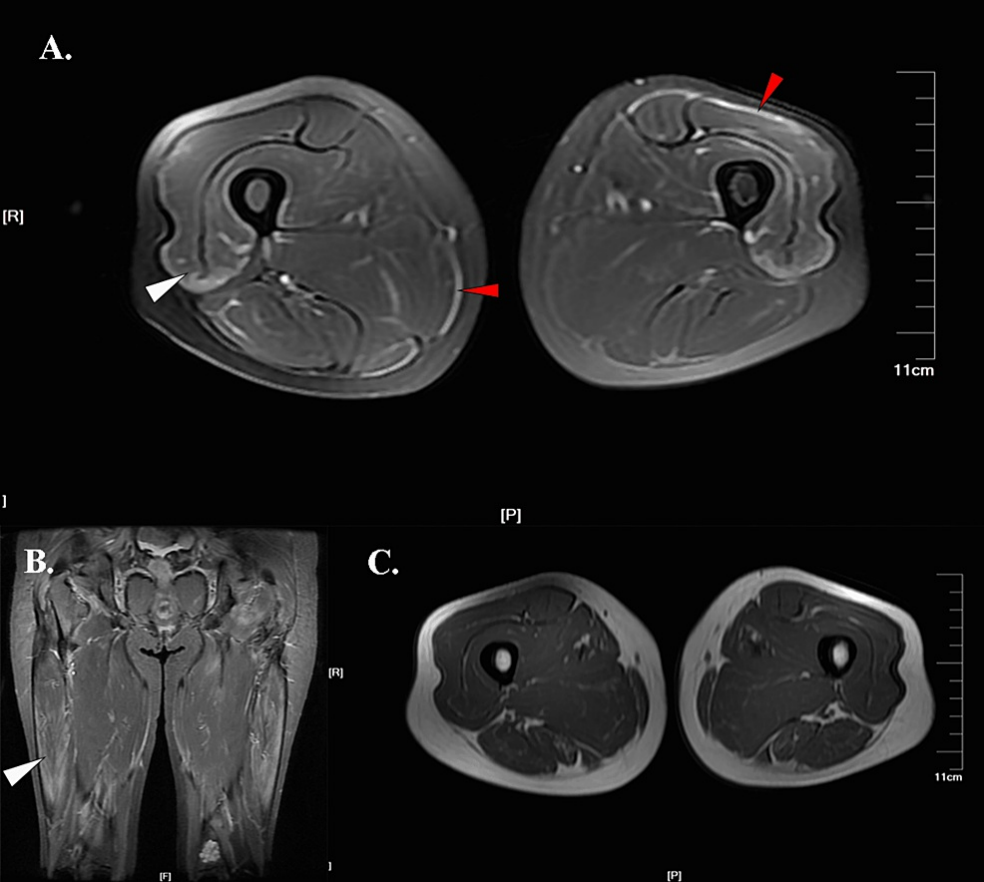
Thigh muscle MRI images of Case 3 A. An axial T2 fs sequence image showed fascia involvement (red arrows) and peripheral honeycomb edema signal of the vastus lateralis (white arrow). B. A coronal STIR sequence image showed a vastus lateralis edema signal (white arrow). C. An axial T1 sequence image showed no fatty infiltration. Abbreviations: T2 fs, T2-weighted fat saturation; STIR, short time of inversion recovery

A 60-year-old postmenopausal female started to develop a skin rash on the forehead, cheeks, and neck for six months. After receiving treatment for dermatitis, the symptoms did not improve. Two months later, she gradually felt facial swelling and difficulty climbing. She presented to our hospital with Gottron’s papules, heliotrope rash, V sign over the anterior neck, dysphagia, and grade IV muscle strength. With significantly elevated tumor biomarkers, she underwent a series of cancer screening tests, including nasopharyngeal cancer antibody detection, gastrointestinal X-ray, head CT scan, and gynecological ultrasound. Then, the patient was transferred to the gynecology department for laparoscopy with pathology results of high-grade serous adenocarcinoma of the left adnexal ovary. Thus, the diagnosis of dermatomyositis and ovarian cancer was confirmed. With the treatment of intravenous prednisolone injection (40 mg/d for seven days) and oral intake of hydroxychloroquine sulfate tablets (0.2 g BID), her muscle strength improved to grade V. The musculoskeletal MRI demonstrated fascia hyperintensity and a symmetrical peripheral honeycomb signal pattern on the vastus lateralis, as shown in Figures 3A-3B. No high-intensity signal was found in subcutaneous adipose tissue, adductors, and posterior thigh compartments. No obvious fatty infiltration was seen on the T1 sequence (Figure 3C).

## Discussion

In this case series report, we described the MRI edema signal features of three dermatomyositis (DM) patients who were diagnosed with ovarian cancer. Fascia involvement along with a typical symmetrical peripheral honeycomb hyperintensity pattern of the vastus lateralis was found in all three patients.

Dermatomyositis (DM) is an autoimmune disease that predominantly affects skeletal muscle, presenting with muscle inflammation and other systemic manifestations, including internal malignancy. The diagnosis is mainly based on symptoms, increased creatine kinase levels, electromyogram findings, and muscle biopsy results. Musculoskeletal MRI is of great significance in assessing the severity of disease and guiding the location for muscle biopsy. Among the information MRI offers, researchers highlight the edema/inflammation signal evaluated on T2 fs/STIR sequences. Muscle edema was found to be associated with the intensity of the inflammatory infiltrate in muscle biopsies and correlated with disease activity [10-11].

MRI for the classification of inflammatory myopathies has been increasingly valued by researchers in recent years. Early in 2019, Ukichi et al. described MRI features for DM as subcutaneous HSI (high signal intensity), fascial HSI, and honeycomb pattern [8]. In 2021, Spalkit et al. concluded the muscle MRI findings of DM as symmetric diffuse intramuscular hyperintensity involving the anterior, posterior, and medial compartments of the thigh, preferably the quadriceps femoris [12]. By comparison with polymyositis and inclusion body myositis patients, Pilania et al. summarized MRI features of DM as subcutaneous and fascial edema, frequent quadriceps involvement, and peripheral distribution/honeycomb pattern [13]. In our report, we found one patient with subcutaneous adipose tissue affected, and all three patients had fascia and thigh muscle involvement, which corresponded to previous reports. We observed that all the patients demonstrated peripheral honeycomb edema signals, mostly in the vastus lateralis. The underlying mechanism of DM is complement-mediated vasculopathy of the small vessels; thus, the peripheral honeycomb edema signal could be explained by predominantly perivascular and perifascicular inflammation [14].

MRI edema signals can also function as prognostic predictors. In 2011, Ladd et al. illustrated abnormal subcutaneous adipose signals associated with clinical outcomes in juvenile dermatomyositis patients while muscle or fascia involvement was not correlated [9]. Recently, Karino et al. defined myofascial-dominant involvement with whole-body MRI as a risk factor for rapidly progressive interstitial lung disease, another complication of DM with high mortality [15]. As a possible paraneoplastic manifestation, there are no studies that have analyzed the MRI features of dermatomyositis patients with cancer prognosis. Previously, few cases reported DM with nivolumab treatment [16] or after tumor removal [17], while in this study, we focused on ovarian cancer and excluded patients diagnosed with cancer before DM to eliminate the possible influence of immune checkpoint inhibitors. DM was considered a key for ovarian cancer diagnosis, Field et al. reported two cases that shared BRCA mutations [18]. More importantly, ovarian cancer is one of the three types of cancer that are associated with DM before diagnosis [19] so more clues are needed to identify the underlying cancer risk. Our observation is that MRI reticular edema signals in the marginal zone of the vastus lateralis have the potential to be an alert for physicians identifying DM patients who need more detailed examinations.

Cancer is most often diagnosed in the first year after the diagnosis of dermatomyositis [20]. The underlying mechanism for the linkage between cancer and DM remains unclarified. CA12-5 and gynecological ultrasound are helpful to identify ovarian cancer; however, as in Case 1, both examinations remained normal even with cancer metastasis. Therefore, as a widely used tool, our findings on thigh muscle MRI have important implications to assist clinicians in identifying patients with potential ovarian cancer risk.

## Conclusions

To understand whether dermatomyositis patients complicated with cancer demonstrate specific muscle MRI features, here, we presented the MRI edema features of three patients diagnosed with ovarian cancer after the diagnosis of DM. Interestingly, we found that apart from adipose tissue and fascia involvement, a honeycomb signal distributed to the peripheral area of the vastus lateralis was observed in all patients. Further study with a large cohort is needed to verify whether this feature could be a potential prognostic predictor. Our report has important implications for clinicians to identify patients with dermatomyositis at potential ovarian cancer risk.
